# Occurrence modes of gold and relevant analytical methodologies

**DOI:** 10.1039/d5ra08103c

**Published:** 2026-03-18

**Authors:** Dong Li, Xiao Zhang, LiJuan Zhang, PengDa Fang, Liang Feng, JinCheng Wang, ZiJian Zhang

**Affiliations:** a China Geological Survey Langfang Integrated Natural Resources Survey Center Tianjin 300301 China

## Abstract

Gold chemical phase analysis is a key technique for determining the occurrence modes of gold in ores and guiding mineral processing decisions. Research in this field is generally divided into physical and chemical analysis. Chemical phase analysis operates on the principle of sequential selective extraction, using specific solvent sequences (*e.g.*, iodine solution, cyanide, sodium thiosulfate) to selectively dissolve different host minerals, thereby enabling the quantitative determination of phases such as free gold, sulfide-occluded gold, and silicate-occluded gold. For measuring gold in different phases, methods including amalgamation-iodine leaching, amalgamation-thiourea-iodine leaching, amalgamation-bromine-iodine leaching, and cyanidation have been established. However, current methods face several challenges: reliance on toxic reagents like mercury and cyanide poses environmental and safety risks; analytical procedures are complex, and accuracy is often compromised by the complexity of gold occurrence, insufficient solvent selectivity, and the preg-robbing effect; furthermore, pretreatment is costly and prone to causing pollution. Future research should focus on developing green, low-toxicity leaching reagents and fostering closer integration between precise phase characterization and efficient pretreatment technologies. This will enhance analytical accuracy, efficiency, and environmental friendliness, thereby supporting the green and efficient utilisation of refractory gold resources.

## Abstract 2

Gold chemical phase analysis quantifies different gold occurrence states through selective dissolution to assess ore processability.

## Graphical abstract

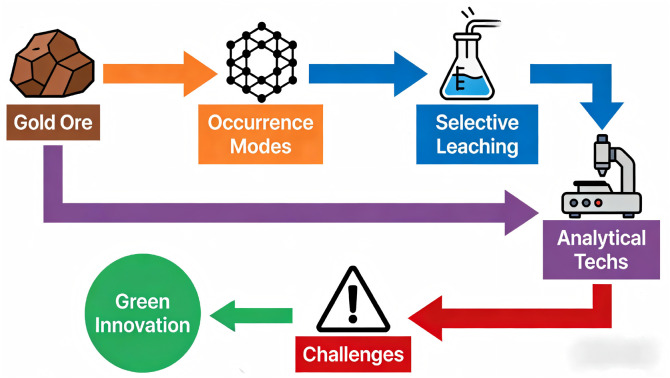

## 1. Introduction

Gold, a valuable precious metal, is highly valued for its exceptional corrosion resistance, electrical conductivity, and thermal conductivity. The distribution, migration, and enrichment of gold within the Earth's system are fundamentally controlled by its geochemical properties, which determine its natural occurrence and economic extractability. Owing to its high ionization energy, electronegativity, and standard redox potential, gold is chemically inert and predominantly exists in its native (metallic) form. However, under specific physicochemical conditions—such as in hydrothermal systems with high oxygen fugacity—gold can be oxidized to form ionic species (Au^+^ and Au^3+^) and become mobile in fluids. These gold ions, exhibiting both siderophilic and chalcophilic affinities, readily form stable soluble complexes with ligands (*e.g.*, Cl^−^, HS^−^, S^2−^), enabling their long-distance transport in crustal fluids. The eventual precipitation and enrichment of gold to form economic deposits occur when changes in physicochemical conditions (*e.g.*, temperature, pressure, pH, or ligand activity) destabilize these complexes, leading to their dissociation and the reduction of gold.^1–4^

With the continuous exploitation of gold resources, easily extractable gold ores are diminishing, while refractory gold resources have demonstrated increasing development potential and practical importance. In such ores, gold often occurs as “invisible” fine-grained particles locked within minerals such as pyrite and arsenopyrite, or associated with carbonaceous matter, as seen in deposits like the South Deep Gold Mine in South Africa and the Mount Morgan Gold–Copper Mine in Australia. Currently, refractory gold ores constitute a substantial and increasing share of global gold production—approximately 30%—with expectations for this proportion to rise further in the future. In this context, a thorough understanding of the modes of occurrence of gold in ores has become essential for the effective development and utilization of refractory gold resources.

## 2. Phase classification of gold

Gold phase analysis is a fundamental technique used to determine the specific occurrence modes of gold in geological samples such as ores, concentrates, and tailings through chemical or physical means. Based on analytical principles, gold phase analysis methods can be broadly divided into two categories: physical analysis and chemical analysis.

Physical analysis employs techniques such as microscopic observation, scanning electron microscopy (SEM), electron probe microanalysis (EPMA), and X-ray diffraction (XRD) to directly identify and characterize the form and distribution of gold in samples.^5^ In contrast, chemical analysis leverages the differential solubility of gold and its host minerals in specific leaching reagents. This approach involves selective dissolution, separation, and quantitative measurement of gold across various phases. Alternatively, process mineralogical attributes of gold can be inferred through mathematical methods such as regression analysis, based on correlations between gold and associated elements.^6^

Thus, gold phase analysis not only provides theoretical insights into gold mineralization processes and supports mineral exploration but also delivers essential data for optimizing mineral processing and extractive metallurgy, thereby improving resource utilization efficiency.

(1) The occurrence modes of gold in ores are diverse and complex, and their accurate identification represents the primary objective of phase analysis. These modes can be generally classified as follows:

(2) Gold minerals: predominantly include native gold and electrum, which constitute independent mineral phases and represent the most economically significant forms.

(3) Colloidal gold: extremely fine gold particles occurring in colloidal form, often adsorbed on mineral surfaces or within microfractures.

(4) Lattice gold: gold present in atomic or ionic form substituting for other elements within the crystal structure of host minerals. This form is relatively uncommon and is generally not a primary target in routine phase analysis.

(5) Adsorbed gold: refers to ionic or nanoparticulate gold adsorbed onto mineral surfaces, such as those of clay minerals.

(6) Organic gold: gold occurring in the form of organometallic complexes, often grouped together with adsorbed gold under the category of organically bound or carbonaceous gold.^7^

In practice, gold phase classification must be tailored to sample characteristics. Commonly recognized chemical phases include: free gold (native Gold), gold in intergrowths, carbonate-bound gold, sulfide-encapsulated gold (*e.g.*, in pyrite or sphalerite), silicate/quartz-encapsulated gold, limonite-encapsulated gold.

A range of chemical phase analysis methods has been developed to target these different forms, including: amalgamation-iodine leaching, amalgamation-thiourea-iodine leaching, amalgamation-bromine–iodine leaching, cyanidation (cyanide leaching), sodium thiosulfate-sodium sulfite leaching, iodine–bromine-perchloric acid-hydrochloric acid leaching, hydrochloric acid-EDTA-H_2_O_2_ leaching.^7–17^

## 3. Principles and methods of selective chemical extraction for gold phase analysis

The fundamental principle of chemical phase analysis is “stepwise selective extraction.” This process involves using a series of chemical reagents with specific dissolution capabilities and conditions to sequentially dissolve the host minerals, thereby progressively releasing gold from different phases, followed by the determination of its content in each phase. Various chemical extraction analysis methods have been established for targeting different gold phases.^9,10,12,16,18,19^

### 3.1 Amalgamation-iodine leaching method^20,21^

This method categorizes gold into four chemical phases: free native gold, gold in intergrowths, gold in sulfides, and gold in other minerals. The analytical procedure for each phase is as follows. Free native gold is separated using the amalgamation method. This technique is based on the property of gold to form gold amalgam (AuHg_2_) with mercury. Under suitable conditions, mercury selectively wets and dissolves the surface of native gold, thereby achieving its separation from other minerals.Au + 2Hg → AuHg_2_

Intergrown gold is selectively extracted *via* an iodine–potassium iodide solution. The principle involves the formation of the I_3_^−^ complex ion from iodine in the presence of iodide ions. This complex subsequently reacts with gold to form soluble complexes such as [AuI_2_]^−^ or [AuI_4_]^−^. This process dissolves gold exposed at mineral surfaces or along intergrowth boundaries, while leaving the main structure of the host minerals largely intact.2Au + I_2_ + 2I^−^ → 2[AuI_2_]^−^2Au + 3I_2_ + 2I^−^ → 2[AuI_4_]^−^

The mineral residue, after removal of the intergrown gold, is roasted at a specific temperature. This causes the decomposition and oxidation of sulphides and the disintegration of their structure, thereby releasing fine encapsulated gold particles. The roasted product is then subjected to a second extraction with the iodine–potassium iodide solution (the reaction mechanism is identical to that for intergrown gold) to determine the gold content within the sulphide phase.
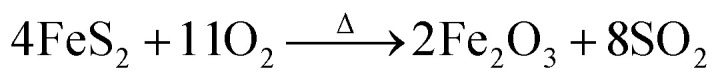

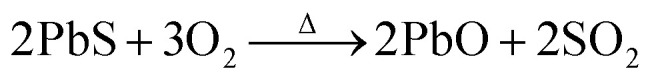


Finally, the gold contained within other minerals (primarily silicate and carbonate gangue minerals) is quantified by strongly digesting the residual material after the sequential removal of the aforementioned phases. The determination of total gold content is accomplished by a combined method: sample dissolution with aqua regia (1 + 1) followed by activated carbon adsorption, and digestion of the residue with a mixed acid of aqua regia–perchloric acid–hydrofluoric acid, also followed by activated carbon adsorption. Aqua regia (a mixture of concentrated hydrochloric and nitric acids) provides strong oxidising power and chloride ions for complexation, effectively dissolving metallic gold. Hydrofluoric acid specifically reacts with the silicate tetrahedra in silicate minerals, generating volatile silicon tetrafluoride (SiF_4_) or soluble fluoro silicates. This thoroughly disrupts the lattice structure of quartz and silicates, ensuring the complete release of occluded gold into solution for subsequent determination.Au + 3NO_3_^−^ + 6H^+^ + 4Cl^−^ → [AuCl_4_]^−^ + 3NO_2_↑ + 3H_2_O

This method offers the advantage of clear phase delineation. By employing distinct chemical approaches—amalgamation, selective extraction with iodine–potassium iodide, roasting to release encapsulated gold, and combined acid digestion—it effectively distinguishes and quantifies free gold, intergrown gold, sulphide-occluded gold, and gangue-mineral-locked gold. This capability is of significant value for elucidating gold occurrence and distribution and for guiding mineral processing and extraction strategies. However, the procedure also has drawbacks: it is relatively complex and time-consuming, involves hazardous reagents such as mercury and hydrofluoric acid, and consequently requires stringent laboratory safety measures. Moreover, the stability of the iodine–iodide system and the potential for gold volatilisation during roasting may affect the accuracy of determination. These issues must be mitigated through strict process control and appropriate blank corrections.

**Fig. 1:**
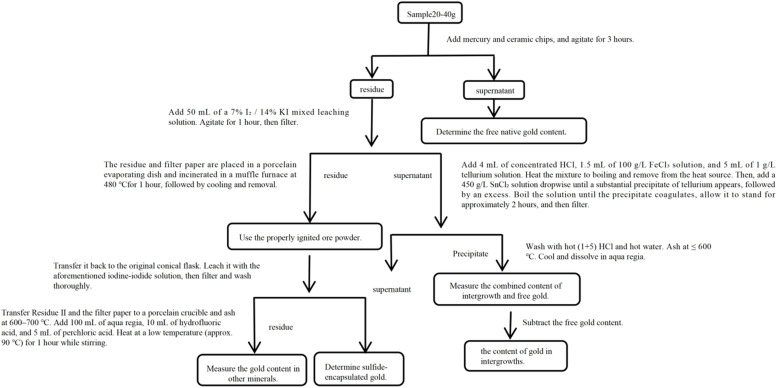
Determination of gold phases in gold ore by the amalgamation-iodine leaching method.

The complete operational procedure is summarized in Fig. 1.

### 3.2 Amalgamation-thiourea-iodine leaching method^5^

The chemical phase analysis of gold is divided into six distinct phases: free native gold, intergrown gold, galena-occluded gold, sphalerite-occluded gold, pyrite-occluded gold, and quartz- and silicate-locked gold. The determination of free gold is based on the amalgamation method, which achieves separation *via* the alloying reaction whereby gold forms gold amalgam (primarily AuHg_2_) upon contact with liquid mercury.Au + 2Hg → AuHg_2_

Intergrown gold is determined using a thiourea–sulphuric acid–ammonium ferric sulphate solution system. In this system, ammonium ferric sulphate acts as an oxidant to oxidise gold to Au(i), while thiourea acts as a complexing agent to form the stable cationic complex [Au(SC(NH_2_)_2_)_2_]^+^, which enters the solution.Au + 2SC(NH_2_)_2_ + Fe^3+^ → (Au(SC(NH_2_)_2_)_2_)^+^ + Fe^2+^

Galena-occluded gold is determined with an iodine–potassium iodide solution. Iodine forms the I_3_^−^ complex ion in the presence of iodide ions, which selectively dissolves the galena, thereby exposing the occluded gold particles. The gold is subsequently oxidised to form soluble [AuI_2_]^−^ and enters the solution.PbS + I_3_^−^ + 2H^+^ → Pb^2+^ + S + 3I^−^ + H_2_

Sphalerite-occluded gold is determined by first leaching its structure with hydrochloric acid, followed by extraction and determination of the released metallic gold using the thiourea–sulphuric acid–ammonium ferric sulphate solution.ZnS + HCl → ZnCl_2_ + H_2_S

Pyrite-occluded gold is determined by initially oxidising and decomposing the mineral lattice with nitric acid to release the fine gold particles, which are then extracted and determined using the same thiourea-based system.FeS_2_ + 5HNO_3_ → Fe^3+^ + 2SO_4_^2−^ + 5NO_2_↑ + H^+^ + H_2_O

Finally, quartz- and silicate-locked gold is treated with a mixed aqua regia–hydrofluoric acid solution. Aqua regia dissolves the gold, while hydrofluoric acid destroys the silicate structures, ensuring the complete release of the locked gold into the solution for determination.Au + 3NO_3_^−^ + 6H^+^ + 4Cl^−^ → [AuCl_4_]^−^ + 3NO_2_↑ + 3H_2_O

Its advantage lies in the more refined delineation of phases, particularly through the differentiation of major sulphide carriers (galena, sphalerite, and pyrite). By adopting targeted decomposition approaches—such as iodine–potassium iodide treatment, hydrochloric acid pre-leaching, and nitric acid oxidation—according to the distinct chemical properties of each mineral, followed by gold extraction with either thiourea or iodine systems, the analytical results can more accurately reflect the specific distribution patterns of gold in complex polymetallic ores. This offers enhanced guidance for process mineralogy studies and the optimisation of extraction and processing strategies.

**Fig. 2:**
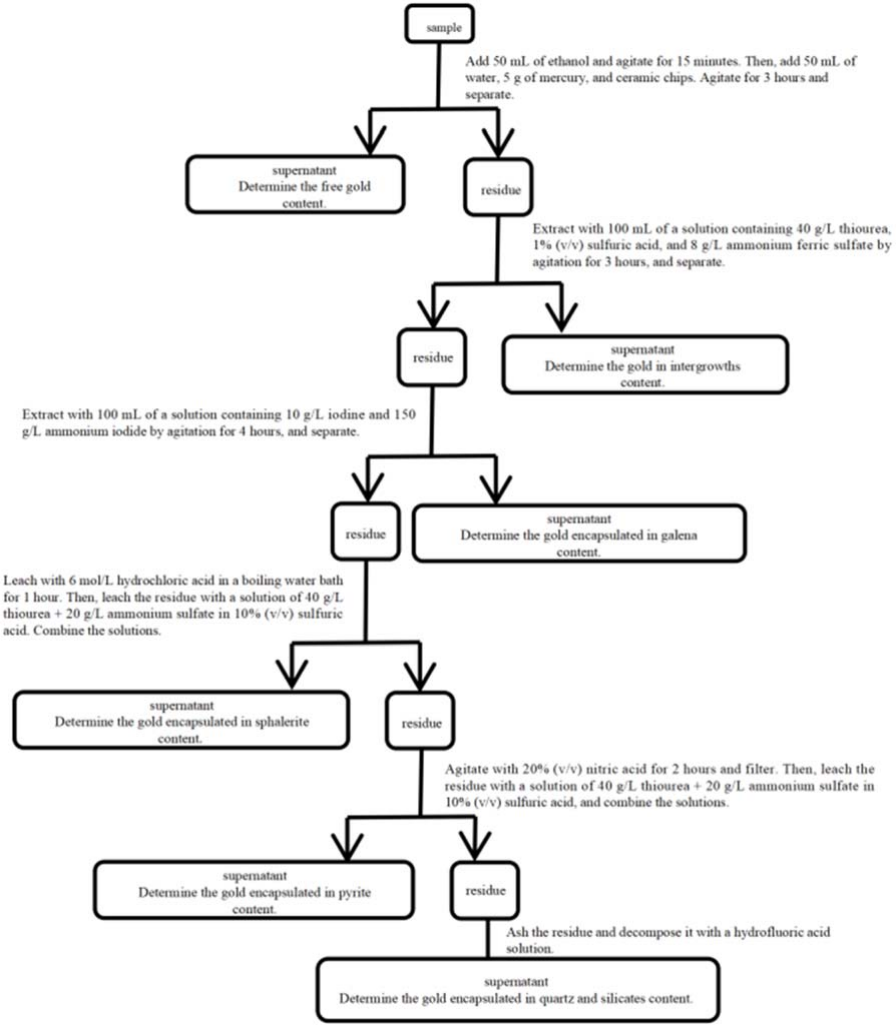
Determination of gold phases in gold ore by the amalgamation-thiourea-iodine leaching method.

However, its drawbacks are also more pronounced: the procedure is extremely lengthy and complex, increasing the risk of interference between successive steps. In particular, it is difficult to perfectly balance thoroughness and selectivity during sequential dissolution, which may lead to re-dissolution or loss of phases. Steps such as the oxidation of pyrite with nitric acid involve severe conditions that may promote gold encapsulation or volatilisation. The alternating use of multiple reagent systems (thiourea, iodine solutions, strong acids, *etc.*) demands exceptionally high operational precision and safety measures, while also significantly increasing both cost and time consumption (Fig. 2).

### 3.3 Amalgamation-bromine-iodine leaching method^5^

The method for determining free gold and intergrown gold in the amalgamation-bromine-iodine extraction procedure (Yao Jiyang and Bao Meiling, 1992) is the same as that used in the amalgamation-iodine extraction method. Sulphide-occluded gold is extracted using a bromine-methanol solution, in which bromine acts as a strong oxidant to effectively destroy the sulphide lattice, while methanol provides a suitable reaction medium. Upon decomposition of the mineral, the occluded gold is oxidised to the soluble complex ion [AuBr_4_]^−^ and enters the solution.2Au + 3Br_2_ + 2Br^−^ → 2[AuBr_4_]^−^

Pyrite-occluded gold is decomposed with aqua regia (HCl + HNO_3_) after a hydrochloric acid pretreatment to remove residual reagents. Nitric acid thoroughly disrupts the pyrite structure, and the released gold forms the stable complex [AuCl_4_]^−^ with nascent chloride ions, thereby enabling its determination.Au + NO_3_^−^ + 4H^+^ + 4Cl^−^ → [AuCl_4_]^−^ + NO↑ + 2H_2_O

For quartz- and silicate-locked gold, a mixed aqua regia-hydrofluoric acid solution is employed. Hydrofluoric acid specifically attacks the siloxane framework (generating SiF_4_), while aqua regia simultaneously oxidises and complexes the released gold.

**Fig. 3:**
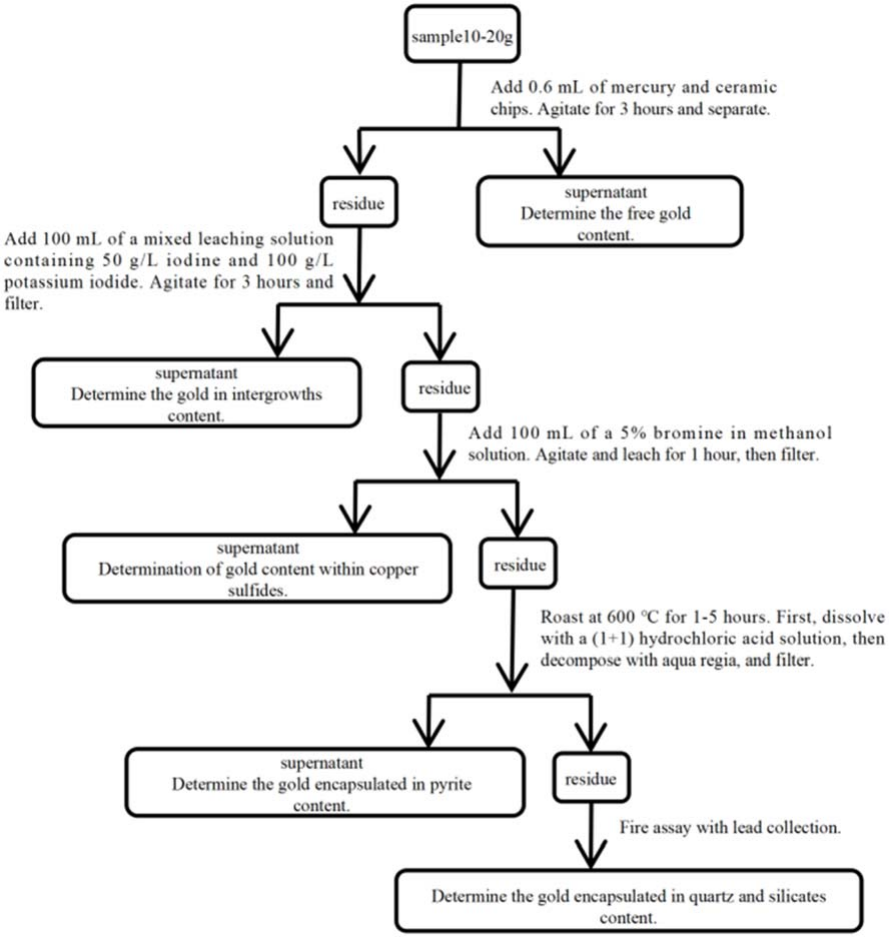
Determination of gold phases in gold ore by the amalgamation-bromine–iodine leaching method.

The advantage of this method lies in its clear phase delineation and targeted specificity. However, it also exhibits notable drawbacks: the procedure involves multiple highly hazardous reagents, including mercury, bromine, aqua regia, and hydrofluoric acid, resulting in cumbersome operations and stringent safety requirements. Multi-step separations may introduce cross-contamination or lead to the re-dissolution of phases, compromising measurement accuracy. Moreover, the reagents are costly and pose a significant environmental burden (Fig. 3).

### 3.4 Cyanidation method

The cyanidation method (Yao Jiyang and Bao Meiling, 1992) employs sodium cyanide or potassium cyanide solution to extract and determine gold in its free state and within sulphide minerals. The principle is that, under aerobic conditions, cyanide ions (CN^−^) react with gold to form the stable dicyanoaurate complex ion ([Au(CN)_2_]^−^), which passes into solution.4Au + 8CN^−^ + O_2_ + 2H_2_O → 4[Au(CN)_2_]^−^ + 4OH^−^

Subsequently, the extraction residue is decomposed using an aqua regia–hydrofluoric acid mixed solution to determine quartz- and silicate-locked gold. Here, hydrofluoric acid (HF) specifically disrupts the siloxane framework of silicates and quartz, generating volatile SiF_4_, while aqua regia (HCl + HNO_3_) provides a strongly oxidising and complexing environment, dissolving the released gold and converting it into the [AuCl_4_]^−^ complex, thereby enabling the quantitative determination of gold in this phase.

The advantages of this method are: high efficiency and good selectivity in cyanide leaching, which effectively dissolves exposed or finely encapsulated gold in free and sulphide forms, and a procedure that is more streamlined than the aforementioned sequential methods. The subsequent aqua regia–hydrofluoric acid treatment ensures complete decomposition of silicates, guaranteeing the full release and determination of locked gold. However, the method has significant drawbacks: the use of highly toxic cyanides poses serious challenges to laboratory safety, waste disposal, and environmental security, limiting its widespread application. Furthermore, cyanide leaching combines gold from different sulphide minerals into a single phase, failing to distinguish between carriers such as galena, sphalerite, and pyrite, thereby reducing the granularity of mineralogical information obtained.

### 3.5 Sodium thiosulfate-sodium sulfite leaching method^22^

The sodium thiosulphate-sodium sulphite extraction method (Zheng Dazhong and Zheng Ruofeng) sequentially determines gold in four distinct phases: exposed and semi-exposed gold, carbonate-occluded gold, sulphide-occluded gold, and quartz- and silicate-locked gold. Exposed and semi-exposed gold is directly dissolved in the sodium thiosulphate-sodium sulphite solution *via* complexation by thiosulphate ions (S_2_O_3_^2−^), forming the stable anionic complex [Au(S_2_O_3_)_2_]^3−^.2Au + 4S_2_O_3_^2−^ + ½O_2_ + H_2_O → 2[Au(S_2_O_3_)_2_]^3−^ + 2OH^−^

Carbonate-occluded gold is first released by decomposing the carbonate minerals with hydrochloric acid. The resultant material is then neutralised and rendered alkaline with concentrated ammonia solution, after which the liberated gold is extracted using the sodium thiosulphate-sodium sulphite solution. Sulphide-occluded gold is extracted with a mixed solution of sodium thiosulphate, sodium sulphite, and copper–ammonia complex. Here, the tetraamminecopper(ii) ion ([Cu(NH_3_)_4_]^2+^) acts as both a catalyst and a mild oxidant. It promotes the oxidation and dissolution of the sulphide surfaces while accelerating the complexation-dissolution of gold *via* the Cu(ii)/Cu(i) redox cycle.Au + S_2_O_3_^2−^ + [Cu(NH_3_)_4_]^2+^ → [Au(S_2_O_3_)_2_]^3−^ + [Cu(S_2_O_3_)]^−^ + 4NH_3_

The final residue is decomposed with aqua regia-hydrofluoric acid to determine quartz- and silicate-locked gold. Hydrofluoric acid destroys the silicon-oxygen framework, while aqua regia oxidises and complexes the gold, ensuring its complete dissolution and determination.

The method selectively extracts gold from different phases sequentially through a sodium thiosulphate–sodium sulphite system. Its advantages include the avoidance of highly toxic reagents such as cyanide and mercury, resulting in significantly improved environmental compatibility. Furthermore, it incorporates a dedicated acid digestion–neutralisation pre-treatment step for carbonate-occluded gold and employs a copper–ammonia complex as a catalyst to mildly yet effectively extract sulphide-occluded gold, offering reasonable phase delineation and good specificity.

**Fig. 4:**
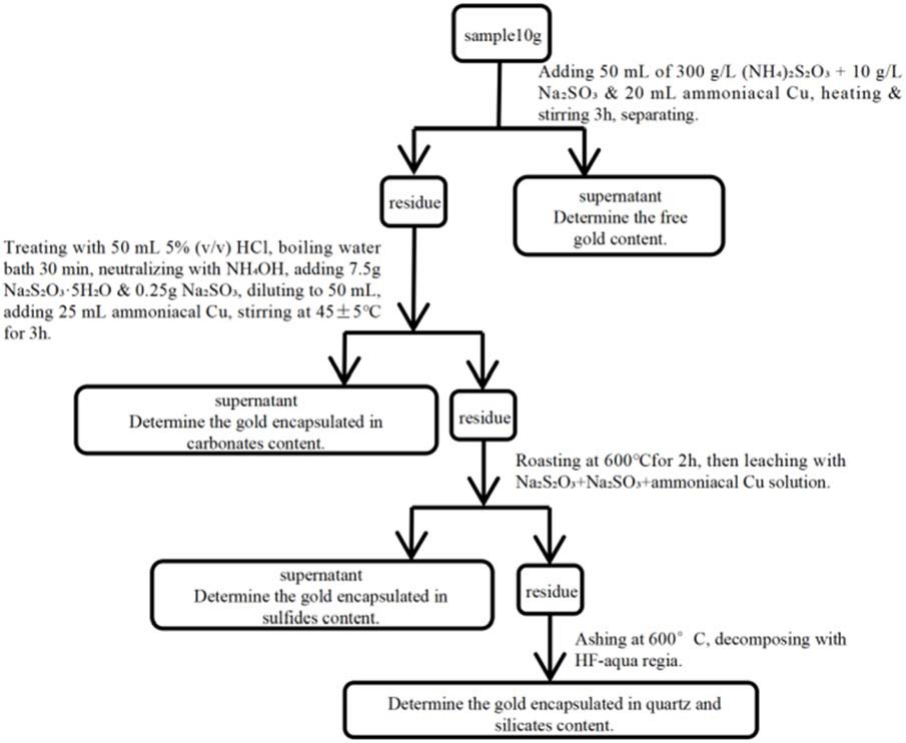
Determination of gold phases in gold ore by the sodium thiosulfate-sodium sulfite leaching method.

However, the method also presents notable drawbacks. The sodium thiosulphate system is insufficiently stable in air and prone to decomposition, which can lead to fluctuations in gold extraction efficiency and necessitates strict control of experimental conditions. The procedure remains relatively complex, involving multiple steps of pH adjustment and phase separation, which complicates operation and may introduce errors. Additionally, the method combines gold from various sulphides into a single determination, failing to differentiate between carrier minerals and consequently losing some process mineralogical information (Fig. 4).

### 3.6 Iodine-bromine-perchloric acid-hydrochloric acid leaching method^23^

According to the analytical procedure for different gold phases in the iodine–bromine–perchloric acid–hydrochloric acid extraction method (Xiao Jiang, 2020), the process is as follows: exposed and semi-exposed gold is extracted using an iodine–ammonium iodide solution, in which iodine (I_2_) acts as a mild oxidant to oxidise gold and form the soluble complex [AuI_2_]^−^, within a medium provided by iodide ions (I^−^) and ammonium ions (NH_4_^+^) from ammonium iodide.

Carbonate-occluded gold is treated with a perchloric acid solution. Leveraging its strong acidity and oxidising power, perchloric acid rapidly decomposes carbonate minerals, exposing the occluded gold which is then extracted and determined within the acidic, oxidising environment. Copper, lead, and zinc sulphide-occluded gold is extracted with a bromine–methanol solution. Bromine serves as a selective oxidising solvent in the methanol medium to decompose the sulphide lattices, and the released gold is oxidised to [AuBr_4_]^−^, entering the solution. Limonite-occluded gold is treated sequentially with a hydrochloric acid–stannous chloride aqueous solution, a stannous chloride solution, and finally an iodine–potassium iodide solution. Hydrochloric acid and the reduced tin salts jointly disrupt the limonite structure and maintain the gold in an active state, before the exposed gold is ultimately leached by the iodine–potassium iodide solution, thereby overcoming the encapsulation and shielding effect of limonite.FeOOH + 3H^+^ + Sn^2+^ → Fe^2+^ + Sn^4+^ + 2H_2_O

Pyrite-occluded gold is treated, under specific conditions, with an iodine–potassium iodide solution. Through mild oxidation, this step gradually releases and dissolves gold located near the surface or within micro-fractures. Finally, the residue is decomposed with an aqua regia–hydrofluoric acid mixed solution. Hydrofluoric acid completely destroys the silicon–oxygen framework of quartz and silicate minerals, while aqua regia simultaneously oxidises and complexes the released gold, enabling the complete determination of quartz- and silicate-locked gold.

Its advantage lies in the extremely refined delineation of phases. Notably, it innovatively treats limonite-occluded gold as an independent phase and incorporates a dedicated procedure involving hydrochloric acid–stannous chloride pre-treatment followed by iodine-solution leaching, which significantly enhances the analytical capability for determining gold occurrence in oxidised ores. The selection of reagents at each step is well-targeted: for example, iodine solutions enable mild oxidation of exposed gold and near-surface gold in pyrite, bromine-methanol solutions effectively decompose copper-lead-zinc sulphides, and perchloric acid rapidly dissolves carbonates, reflecting a highly chemically logical system design.

**Fig. 5:**
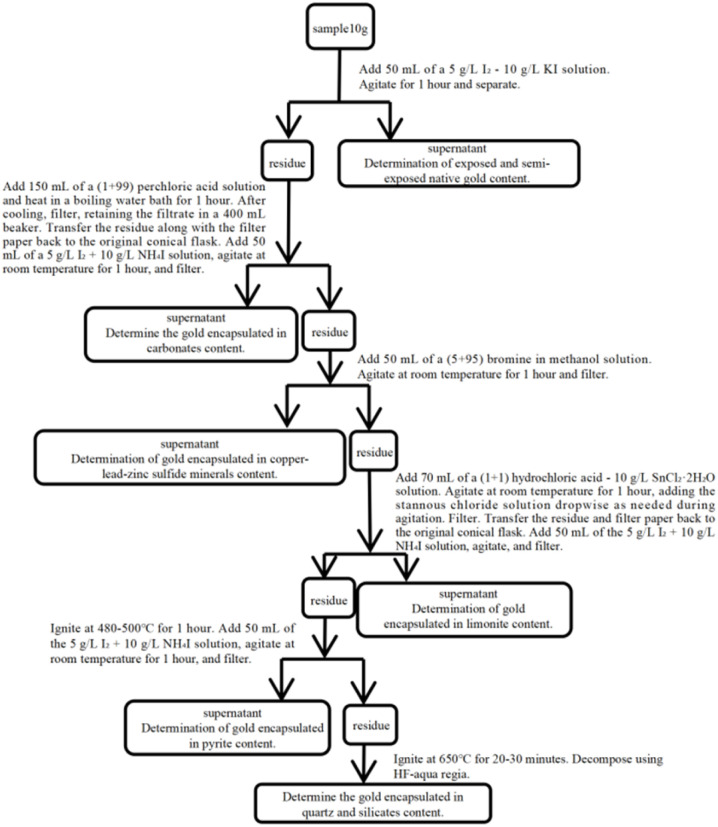
Determination of gold phases in gold ore by the iodine–bromine-perchloric acid-hydrochloric acid leaching method.

However, the drawbacks of the method are also pronounced: the procedure is notably complex and lengthy, involving at least six distinct chemical systems (iodine, bromine, perchloric acid, tin salts, aqua regia-hydrofluoric acid, *etc.*). The sequential dependence among steps imposes stringent demands on operational precision and condition control, making the process susceptible to cross-phase contamination due to washing residues or overlapping selectivity. Some reagents, such as stannous chloride solutions, are unstable in air, which can compromise method reproducibility. Moreover, the use of highly corrosive and hazardous reagents like hydrofluoric acid and perchloric acid poses a severe challenge to laboratory safety and protective measures (Fig. 5).

Furthermore, based on different industrial requirements, gold phases are categorized into distinct types. The hydrochloric acid–EDTA–H_2_O_2_ extraction method (He Chi, 2022) determines associated gold in the oxidised phase by leaching with a hydrochloric acid solution; determines associated gold in the sulphide phase with an EDTA–H_2_O_2_ solution; determines occluded gold in the gangue phase with a sulphuric acid–hydrofluoric acid solution; determines gold in the telluride phase with a nitric acid solution; and determines native gold by aqua regia extraction. The principle of using the EDTA–H_2_O_2_ solution for determining associated gold in the sulphide phase is based on the oxidation reaction of H_2_O_2_ with sulphides to generate metal cations, which are then complexed by EDTA to form stable, soluble complexes.2FeS_2_ + 15H_2_O_2_ → 2Fe^3+^ + 44SO_4_^2−^ + 2H^+^ + 14H_2_OFe^3+^ + H_2_Y^2−^ → FeY^−^ + 2H^+^

The tartaric acid–potassium sulphide method (Zhang Hua, 2010) is used to determine the antimony–gold phase with a tartaric acid–concentrated aqua regia–potassium sulphide solution. The tartrate ions dissolve antimony ions in the solution, preventing their hydrolysis and precipitation.Sb^5+^ + C_4_H_4_O_6_^2−^ + H_2_O → [SbO(C_4_H_4_O_6_^2−^)]^−^ + 2H^+^

All other methods are considered general procedures: hydrochloric acid solution is used for the oxidised phase; EDTA solution for the sulphide phase; sulphuric acid–hydrofluoric acid solution for the gangue phase; nitric acid solution for the telluride phase; and aqua regia extraction for free gold.

In recent years, the growing demand for processing refractory gold ores—particularly those containing antimony, carbonaceous matter, and other interferents—has intensified research into efficient gold separation technologies. For antimony-bearing gold ores, Celep *et al.* (2011) and Zhang *et al.* (2023) developed a hydrometallurgical process in which antimony is selectively leached from minerals such as stibnite using a sodium sulfide–sodium hydroxide solution, followed by conventional cyanide leaching of gold.^24,25^ This approach effectively mitigates the adverse effects of antimony on subsequent gold extraction during smelting. Building on this, Liu *et al.* (2019) further improved gold recovery from refractory antimony-bearing ores by introducing a sodium sulfate pre-roasting step prior to leaching.^26^ For gold encapsulated in arsenic- and sulfur-bearing minerals such as pyrite and arsenopyrite, Shen *et al.* (2024) demonstrated that adding a mixture of calcium carbonate and sodium carbonate during roasting pretreatment effectively suppresses the formation of a passivation layer on the mineral surface, thereby enhancing gold leachability.^27^ Meanwhile, Liu *et al.* (2024) proposed a combined approach involving sulfuric acid oxidation pretreatment followed by leaching with an ammoniacal copper-thiosulfate solution, achieving efficient gold extraction from refractory arsenic- and sulfur-bearing ores.^28^

## 4. Process for determining total gold content

When determining the total gold content in gold ores, the classical and authoritative method is fire assay, which is internationally recognised as the benchmark method for gold and silver analysis. For routine analysis, atomic absorption spectroscopy and inductively coupled plasma mass spectrometry are also commonly used in conjunction with fire assay or acid digestion methods.

### 4.1 Fire assay

First, a representative gold ore sample is ground to −200 mesh to sufficiently liberate the precious metals. The sample is then thoroughly mixed in a crucible with precisely proportioned fluxes, including litharge (PbO) as a collector, sodium carbonate and borax as fluxes, silica as a slag former, and flour or potassium nitrate to control the redox environment. The mixture is fused in a furnace at 900–1200 °C. During fusion, the sample decomposes, and precious metals such as gold and silver are collected by the reduced metallic lead and settle to form a lead button, while the gangue components form a silicate slag that floats on top. After cooling and separation, the lead button is placed in a cupel and subjected to cupellation at 850–950 °C. The lead is oxidised and absorbed, leaving behind a gold-silver bead. Silver is subsequently dissolved by parting with nitric acid, yielding a pure gold particle. After washing and ignition, the gold particle is weighed, and the gold content (g t^−1^) is finally calculated based on the mass of the gold particle and the sample mass.

### 4.2 Acid digestion method

The acid digestion method (also known as the wet chemical method) for determining gold content in gold ores is currently the most widely used routine analytical technique, particularly suited for the high-throughput testing of medium-to low-grade ores. Its core procedure can be divided into three main stages: sample decomposition, separation and enrichment, and determination. The specific process is briefly described below.

#### 4.2.1 Sample decomposition

A representative sample ground to −200 mesh (typically 10–50 g) is placed in a conical flask or PTFE beaker. Aqua regia is added, and the mixture is heated on a hotplate to dissolve the sample. Aqua regia (typically a 3 : 1 volume ratio of concentrated hydrochloric acid to concentrated nitric acid) generates chlorine and nitrosyl chloride, effectively dissolving free gold and host minerals. It is heated to gentle boiling until the volume is reduced and the sample is completely decomposed (usually requiring 1–2 hours).

#### 4.2.2 Separation and enrichment

The decomposed solution contains a large amount of matrix elements and a small quantity of gold, necessitating selective enrichment of the gold. The most common methods are dynamic adsorption on activated carbon or adsorption on polyurethane foam.

(1) Activated carbon adsorption: the hot solution is filtered through a Buchner funnel containing a column of activated carbon under suction. The gold–chloro complex anions are selectively adsorbed onto the activated carbon. The carbon is then washed with dilute hydrochloric acid and hot water to remove impurities, and finally, the gold-bearing activated carbon is ashed.

(2) Polyurethane foam adsorption: a piece of polyurethane foam is placed in the solution and agitated for adsorption. The gold is enriched into the foam, which is then removed, squeezed dry, and ashed.

#### 4.2.3 Determination

The ash containing the enriched gold is re-dissolved in a small amount of aqua regia, made up to a fixed volume, and prepared as the test solution for final measurement.

## 5. Analytical techniques and methods

In the analytical process for determining gold content *via* leaching methods, the accurate analysis of leachates—a specific and complex matrix—is a critical step. Given that gold concentrations in leachates are typically low and the matrix is complex, Graphite Furnace Atomic Absorption Spectrometry (GFAAS), Inductively Coupled Plasma Mass Spectrometry (ICP-MS), and Atomic Fluorescence Spectrometry (AFS) are the three most commonly used high-sensitivity detection techniques in laboratories. Based on different physical principles, they each have specific strengths in sensitivity, interference resistance, and application scenarios, collectively forming a suite of solutions for various analytical requirements.^29–33^

First, Graphite Furnace Atomic Absorption Spectrometry (GFAAS) is currently the primary technique for determining gold content in leachates in geological laboratories. Its core advantage lies in its high sensitivity, enabling the direct determination of low concentrations at the µg L^−1^ (ppb) level. This is primarily due to the efficient preconcentration and atomization of the analyte within the graphite tube platform. In practical application, optimizing the ashing temperature and using matrix modifiers like palladium salts effectively eliminate interferences from complex matrices such as cyanides and chlorides. Combined with Zeeman background correction, this ensures the accuracy of analytical results, making GFAAS a reliable choice for routine production monitoring and process studies.

Second, Inductively Coupled Plasma Mass Spectrometry (ICP-MS) represents the pinnacle of trace element analysis. It offers extremely high sensitivity, 1–2 orders of magnitude greater than GFAAS (reaching ng L^−1^, ppt level), allowing for the determination of ultra-low concentrations in leachates. Its more powerful capability, however, lies in its simultaneous multi-element analysis. In a single analysis, it can provide accurate contents not only for gold but also for associated or interfering elements like silver, copper, arsenic, and antimony. This is invaluable for studying leaching behavior, assessing process efficiency, and evaluating environmental impact.

Finally, Atomic Fluorescence Spectrometry (AFS) also demonstrates good sensitivity for gold. Although gold is not a typical hydride-forming element and its analytical performance is significantly influenced by reaction conditions, AFS technology itself offers advantages of minimal optical interference and low background noise. When equipped with online preconcentration devices or with optimized hydride generation/cold vapour conditions, effective determination at sub-ppb levels can also be achieved, making it a cost-effective alternative for laboratories with budget constraints (Table 1).

**Table 1:** Comparison of gold determination techniques in gold leachate

Instrumentation technology	Advantages	Disadvantages	Comprehensive analysis
Graphite furnace atomic absorption spectroscopy (GFAAS)	1. High sensitivity: directly measures concentrations at the µg L^−1^ (ppb) level	1. Single-element analysis: only one element can be determined at a resulting in relatively low efficiency	The preferred choice for routine analysis and production monitoring. Ideal for daily high-volume sample analysis, production process monitoring, and process research, it strikes the optimal balance between accuracy, stability, and cost-effectiveness, serving as a reliable foundational tool
	2. Strong interference resistance: effectively overcomes complex matrix interferences such as cyanides and chlorides through optimized ashing temperatures, use of matrix modifiers, and Zeeman background correction	2. Slow analysis speed: the heating and atomization process for each sample requires a certain amount of time	
	3. Mature and reliable technology: highly standardized methodology	3. Graphite tube consumption: as a consumable item, it incurs high usage costs	
Inductively coupled plasma Mass spectrometry (ICP-MS)	1. Exceptionally high sensitivity: detection limits reach the ng L^−1^ (ppt) level, surpassing GFAAS by 1–2 orders of magnitude	1. Extremely high instrument and operational costs: expensive to purchase, maintain, and operate on a daily basis	Leading-edge research and complex problem analysis. Suitable for ultra-low concentration sample determination and comprehensive studies requiring multi-element data support
	2. Simultaneous multi-element analysis: rapidly determines gold alongside multiple associated/interfering elements such as silver, copper, and arsenic in a single assay	2. Matrix effects and interference: mass spectrometry interference is present, requiring more specialized tuning and calibration	
	3. Provides richer data dimensions: offers significant value for studying elemental leaching behavior, process evaluation, and environmental monitoring	3. Complex operation: demands high standards for both laboratory environments and personnel	
Atomic fluorescence spectroscopy (AFS)	1. Minimal optical interference and low background noise: atomic fluorescence emission lines are simple and exhibit good specificity	1. Suboptimal gold analysis: gold is not a typical hydride-generating element, and the method's robustness is inferior to GFAAS.	A cost-effective alternative under specific conditions. Suitable for laboratories with limited budgets that can fully leverage its performance through method development. Can serve as a supplement or replacement for GFAAS, but requires thorough method validation
	2. Relatively low instrument costs: significantly reduced acquisition and operational expenses	2. High sensitivity to conditions: analytical results are highly dependent on optimizing hydride/cold vapor generation conditions, making method development challenging	
	3. Excellent sensitivity under optimized conditions: sub-ppb level detection achievable with online enrichment or optimized reaction conditions	3. Limited applicability: primarily suitable for elements that form hydrides or cold vapors, with poor versatility	

In summary, GFAAS, ICP-MS, and AFS constitute a complementary analytical framework for determining gold in leachates. GFAAS, with its well-balanced performance and operational robustness, serves as the cornerstone for routine analysis. ICP-MS, distinguished by its ultra-low detection limits and powerful multi-element capability, is indispensable for advanced research and resolving complex analytical challenges. AFS offers a cost-effective alternative for specific applications. In practice, the selection of an appropriate method should be guided by the required detection limits, desired data dimensionality, sample throughput, and budgetary considerations to ensure the generation of accurate and efficient data for geological exploration, mineral processing, and environmental assessment.

Chemical phase analysis is not employed in isolation; it is most powerful when integrated with techniques such as optical microscopy, scanning electron microscopy with energy-dispersive X-ray spectroscopy (SEM-EDS), and automated mineralogy systems. These methods provide direct morphological and compositional context—including gold particle size, shape, and intergrowth relationships—thereby serving to validate and complement the quantitative findings of chemical phase analysis.

## 6. Challenges and limitations

The accuracy of gold phase analysis and content determination *via* selective leaching techniques is subject to considerable uncertainty due to a range of complex factors, often introducing systematic errors.^34–36^ These limitations primarily arise from the following sources:

First, the intricate occurrence modes of gold and its incomplete liberation during processing pose a fundamental challenge. Gold in ores, especially fine-grained and sub-microscopic (“invisible”) gold, frequently occurs as encapsulated, fracture-filling, or intergrown particles. At the microscopic scale, the “leaching boundary” of a target phase is often poorly defined. For example, partially locked gold within sulfide or silicate matrices may be gradually released through minor etching of the host mineral by the lixiviant, whereas so-called “exposed” gold might not be fully accessible if coated by thin films of other minerals. This “phase crossover” effect disrupts the expected correlation between leaching efficiency and theoretical phase distribution, leading to misclassification.

Second, the limited selectivity of leaching reagents represents an inherent methodological constraint. Although a solvent is theoretically intended to react only with a specific gold-bearing phase, in practice it may also partially dissolve non-target minerals. Cyanide solutions, for instance, can leach associated copper minerals or sphalerite, while iodide-based reagents may mildly attack certain sulfides. Such non-selective dissolution contributes to overestimating gold in the intended phase. While optimizing concentration, pH, and leaching duration can mitigate this interference, it cannot be fully eliminated.

Third, the absence of globally standardized analytical protocols undermines data comparability. Currently, no uniform operational standards govern key parameters—such as reagent type, concentration, liquid–solid ratio, agitation mode, leaching time, or temperature—across different laboratories. Variations in these procedural details hinder meaningful comparison and consistent interpretation of results, even between batches within the same facility.

Finally, the “preg-robbing” phenomenon during leaching can introduce substantial negative errors. In complex leaching systems, dissolved gold complexes such as Au(CN)_2_^−^ may be re-immobilized through several mechanisms:

Re-adsorption onto activated carbon (naturally present in the ore or inadvertently introduced), carbonaceous matter, or clay minerals; Co-precipitation with or encapsulation by secondary sulfides (*e.g.*, iron sulfides) formed in ores rich in sulfur, arsenic, or organic matter; Reduction and re-precipitation as colloidal gold under insufficiently oxidizing conditions.

These processes ultimately return dissolved gold to the solid phase, leading to significant underestimation of extractable gold in solution and misrepresenting the ore's actual leachability.

In summary, gold quantification by leaching is a dynamic and chemically complex process. The final results are influenced by the interplay of gold's initial occurrence state, the non-ideal behavior of lixiviants, inconsistencies in analytical procedures, and competing side reactions. A thorough understanding of these error sources—coupled with careful experimental design, process control, and data interpretation—is essential for obtaining reliable results and accurately assessing the process mineralogy of gold ores.

## 7. Conclusions

In summary, while a diverse array of chemical methods has been established in the field of gold phase analysis—providing essential support for elucidating gold occurrence and optimizing extraction processes—mainstream chemical phase analysis still faces considerable challenges. Key limitations include:

(1) Significant environmental and health risks due to the reliance on toxic reagents such as mercury, cyanide, and methanol, which endanger operator safety and ecosystems, and conflict with the principles of green mining;

(2) Complex and time-consuming analytical procedures, where multi-step operations increase the potential for operational error;

(3) High costs and environmental constraints associated with pretreatment techniques.

Although roasting effectively liberates encapsulated gold,^26,27^ it is energy-intensive, emits harmful gases such as SO_2_ and As_2_O_3_, and entails high waste treatment costs amid tightening environmental regulations.

Looking ahead, future research should prioritize breakthroughs in the following directions:

(1) Developing green and low-toxicity leaching reagents—such as efficient thiosulfate systems, synergistic halide-based lixiviants, and bioleaching agents—to replace highly toxic chemicals.

(2) Enhancing the integration of precise phase-resolution approaches with tailored pretreatment strategies for refractory gold ores containing antimony, arsenic, or high carbonaceous matter [*e.g.*, 25, 28, 37, 38, 39 and 40].

Addressing these issues will substantially improve the accuracy, efficiency, and environmental compatibility of gold phase analysis, thereby laying a solid foundation for the green and efficient utilization of global refractory gold resources and the sustainable advancement of the gold industry.

## Author contributions

Dong Li was responsible for experimental design, experimental operation, figure preparation, and manuscript writing. Xiao Zhang contributed to experimental operation, figure preparation, and manuscript writing. Pengda Fang participated in experimental design and experimental operation. Lijuan Zhang was involved in experimental operation and manuscript review. Liang Feng contributed to experimental design and manuscript review. Jincheng Wang and Zijian Zhang were responsible for experimental operation.

## Conflicts of interest

There are no conflicts to declare.

## Data Availability

No primary research results, software or code have been included and no new data were generated or analysed as part of this review.
